# Epsin bioactive coating reduced in-stent intimal hyperplasia by promoting early phase reendothelialization and inhibiting smooth muscle cell proliferation

**DOI:** 10.1371/journal.pone.0318019

**Published:** 2025-03-25

**Authors:** Wenxu Zhang, Hao Lin, Zechao Zhu, Kunyuan Zhu, Shijun Bi, Xinyu Yang, Guangzhi Hao, Dandan Gao, Da Huo, Shanshan Chen, Jing Zhao, Meixia Liu, Pengyu Pan, Guobiao Liang

**Affiliations:** 1 Department of Neurosurgery, General hospital of Northern Theater Command, Shenyang, China; 2 Institute of Metal Research, Chinese Academy of Sciences, Shenyang, China; 3 China Medical University, Shenyang, China; Shanghai Jiao Tong University Medical School Affiliated Ruijin Hospital, CHINA

## Abstract

In recent years, interventional surgery has become a treatment for ischemic stroke due to its low risk of injury. However, the occurrence of restenosis hinders the long-term effectiveness and safety of stent implantation. At present, drug-eluting stents mainly prevent the stenosis of drug-eluting stents by inhibiting the proliferation of smooth muscle cells (SMCs). However, these drugs cause damage to endothelial cells (ECs), prevent timely re endothelialization of blood vessels, and increase the risk of late thrombosis and late restenosis. EPS-15-interacting protein 1 (Epsin1)- EPS-15-interacting protein 2 (Epsin2)-shrna coated stents have the potential to promote early endothelialization and inhibit restenosis, which contributes to the candidate development of novel drug coated stents. We found that the expression of Epsin was elevated in the mouse carotid artery ligation model, and the intimal hyperplasia(IH) could be reduced by intervening Epsin. Epsin in cultured endothelial cells was interfered to study proliferation and migration functions, and its role in cocultured endothelial cells and smooth muscle cells was evaluated. In addition, we explored the potential therapeutic benefits of inhibiting Epsin in a porcine model using scaffolds coated with plasmids containing Epsin short hairpin RNA (shRNA). Our study showed that the expression of Epsin1 and Epsin2 was elevated in the proliferative intima of mice, and the inhibition of Epsin reduced the proliferation of neointima in mice. The inhibition of Epsin led to enhanced proliferation and migration of endothelial cells, and maintained a healthy cell membrane potential. In cocultured cells, inhibition of Epsin resulted in reduced proliferation and migration of smooth muscle cells. In a porcine carotid artery model, Epsin shRNA coated scaffolds promoted early re endothelialization and reduced IH. These results suggest that Epsin plays a crucial role in endothelial and smooth muscle cell proliferation and migration functions, and its inhibition may be a potentially effective therapeutic strategy to prevent in stent stenosis.

## Introduction

Presently, drugs such as rapamycin and paclitaxel are used to prevent the stenosis of drug-eluting stents by inhibiting the proliferation and migration of smooth muscle cell [1]. In contrast, these drugs cause long-term damage to the endothelial cell preventing timely reendothelialization of blood vessels and increasing the venture of late thrombosis and late restenosis [2,3]. Additionally, endothelial dysfunction and EC loss contribute to the development of IH [4]. After the stent is inserted, rapid reendothelialization could prevent IH and greatly reduce the risk of late in-stent thrombosis and post-stent stenosis [5]. Moreover, promoting the rapid regeneration of EC and preserving their functionality can indirectly inhibit the proliferation and migration of SMCs, thereby reducing IH [6]. We hope to find a way to promote early endothelialization and inhibit the proliferation of smooth muscle cells after stent implantation, so as to prevent the rise of other risks while inhibiting the occurrence of stenosis, The Epsin protein family, known for its role as a highly conserved, membrane-associated, ubiquitin-bound endocytosis adapter, exhibits high specificity in binding protein selection [7–11]. In vascular ECs, Epsin 1 and Epsin 2 represented functionally overlapping members of this protein family that are expressed in both developing and mature lymphatic systems. When investigated role of Epsin, we should intervened both Epsin 1 and Epsin 2 at the same time. The expression of Epsin1 and Epsin2 exhibits variability across different cell types under distinct pathophysiological conditions [12]. In atherosclerosis, Epsin interacted with IP3R1 in ECs [13] and binded to LRP-1 in macrophages [14], which could provide a potential therapeutic by cell-specific targeting of Epsin. It has been shown that endothelial Epsin deficiency decreased tumor growth via vascular endothelial growth factor receptor (VEGFR) 2 signal [15]. In a mice model of atherosclerosis, Epsin activation contributed to the mitigating of endothelial inflammation and activation of endothelial NF-κB [16]. Epsin has the potential to reduce endothelial inflammation after stent implantation, inhibit neointimal hyperplasia and promote early endothelialization, However, the specific role and mechanism of Epsin in carotid IH remained unclear.

In this paper, the expression of Epsin in a mice model of carotid intimal hyperplasia and the corresponding effects on the function of ECs were investigated. An increase of Epsin expression in mice with carotid stenosis and the degree of stenosis decreased after inhibiting the expression of epsin were found, and its impact on EC proliferation, migration, and mitochondrial function was studied. Furthermore, we performed co-culture experiments to evaluate the proliferation and migration of SMCs exposed to ECs with Epsin knockdown, thus shedding light on the underlying molecular mechanisms. Furthermore, we implanted stents coated with Epsin short hairpin RNA (shRNA) plasmids into porcine carotid arteries to evaluate the influence of Epsin on IH.

## Materials and methods

### Materials

1 ×  1 cm2 round 316L flat plates and 316L stents (provided by Institute of Metal Research, Chinese Academy of Sciences, China) were used for in vivo implantation experiments. Protamine sulfate (Prs) (purchased from Merck KGaA, Germany). RIPA lysis buffer (purchased from Thermo Fisher Scientific, USA). Methanol (obtained from Sinopharm Chemical Reagent, China). ECL luminous fluid (procured from NCM Biotech (China). Dulbecco’s Modified Eagle’s Medium (DMEM) (sourced from Gibco, USA). 1% Sodium pentobarbital (formulated by the Pharmacy of the Northern Theater General Hospital, China). Phosphate-buffered saline (PBS) (supplied by Thermo Fisher Scientific, USA). Trypsin (sourced from Sigma, USA). TritonX-100 (purchased from Promega, USA). Goat anti-mice IgG (procured from Abcam, USA). Goat anti-rabbit IgG (sourced from Abcam, USA). Epsin1 antibody (purchased from Santa Cruz Biotechnology, USA). Epsin2 antibody (procured from Origene, China). matrix metalloproteinase (MMP2) antibody (sourced from Abcam, USA). Drp1 antibody (purchased from Abcam, USA). glyceraldehyde-3-phosphate dehydrogenase (GAPDH) antibody (sourced from Abcam, USA). VEGFA recombinant protein and VEGFR2 antibody (procured from Santa Cruz Biotechnology, USA). extracellular regulated protein kinases (ERK) antibody (sourced from Abcam, USA). PERK antibody (purchased from Abcam, USA). Ki67 antibody (procured from Abcam, USA). Mito-Tracker Green (supplied by Beyotime, China). JC-1 (obtained from Beyotime, China). Epsin1- Small interfering RNA (siRNA), Epsin2-siRNA and Epsin1-Epsin2-shRNA (purchased from Hanheng biology science and technology, China). Mouse aortic smooth muscle cells and human umbilical vein endothelial cells (supplied by iCell Bioscience, China).

### Experimental design

This study consists of five experiments, designed as follows.

#### Experiment 1.

In order to determine whether expression of Epsin1 and Epsin2 changes in ECs after vascular IH, a total of twelve mice were randomly allocated into two distinct groups: sham (n = 6) and IH (carotid artery ligation, n = 6). Immunohistochemical staining and hematoxylin and eosin (HE) was used to detect the Epsin1 and Epsin2 expression and spatial distribution 7 days later.

#### Experiment 2.

For purpose of understand the role of Epsin1 and Epsin2 in ECs proliferation and migration, primary ECs were randomly divided into four groups: siControl, siEpsin1 + siEpsin2, siControl+VEGFA (50μg/ml), and siEpsin1 + siEpsin2 + VEGFA (50μg/ml). VEGF was added to the culture medium for 24 hours. Ki67 expression of in each group was detected by immunofluorescence, and cell migration was measured by scratch test. Additionally, VEGFR2 protein and the phosphorylation level of downstream Erk in each group were analyzed by the Western blot. In order to investigate the effects of Epsin1 and Epsin2 on ECs viability, primary ECs were randomly divided into four groups: siControl, siEpsin1 + siEpsin2, siControl+VEGFA (50μg/ml), and siEpsin1 + siEpsin2 + VEGFA (50μg/ml). After adding VEGF to the culture medium for 24 hours, mitochondrial content changes were observed using MitoTraker staining, With the use of JC-1 staining, mitochondrial membrane potentials were observed, and the expression of Drp1 and mitochondrial dynamin like GTPase (OPA1) in each group was also detected by Western blot analysis

#### Experiment 3.

In order to determine the indirect effect of SMC migration and proliferation by knocking down Epsin1 and Epsin2 in ECs, ECs were randomly divided into four groups: siControl, siEpsin1 + siEpsin2, siControl+VEGFA (50μg/ml) and siEpsin1 + siEpsin2 + VEGFA (50μg/ml). Each group was co-cultured with untreated smooth muscle cells (SMC) through Transwell chamber. After 24 hours, Ki67 expression in SMCs was detected by immunofluorescence, the migration of SMCs was detected by scratch testing.

#### Experiment 4.

For purpose of clarify whether knocking down Episn has a therapeutic effect on hyperproliferation of neointima after stenting. Initially, ECs were affixed to the stent’s surface for the purpose of assessing the transfection efficiency of the coated stent. To verify the smoothness and flatness of the coated stent, the surface of the coated stent was scanned by electron microscopy. Six Bama minipigs were randomly assigned into two groups: the control-RNA stent (n = 3) and the Epsin1-Epsin2-shRNA stent (n = 3). After 7 and 28 days of stent implanting, digital substraction angiography was engaged and the target left carotid arteries were remove for HE staining to observe reendothelialization and stenosis.

### Ethics

All procedures for animals were approved by the Ethics Committee of General hospital of Northern Theater Command and follows the principles of Declaration of Helsinki (Approval No.2023-38).

### Carotid artery IH model in mice

C57BL/6J mice was administered intraperitoneally with 1% sodium pentobarbital (50 mg/kg) using a syringe for anesthetizing. The neck median incision was made, after fully disinfecting, and the left neck subcutaneous tissue was gently separated. We performed isolation and ligation of the left common carotid artery (CCA) using a nylon monofilament suture. In the sham group, the same operations were performed except for the ligation of the artery. After eating for 7 days, the mice were anesthetized with isoflurane/O2 and decapitated, the carotid arteries were harvested and stored in liquid nitrogen or paraformaldehyde for further evaluation [17].

### Immunohistochemistry

Following thorough deparaffinization and rehydration, tissue sections were incubated with an oxidase inhibitor for 10 minutes, washed with PBS for 3 times, added with non-immune animal serum for 10min, incubated with primary antibody overnight, rewarming, added with a drop of biotin labeled secondary antibody, washed with PBS for 3 times, added with Streptomyces avidin peroxidase for 10min, washed for 3 times, and colored with DAB solution [18].

### Cells culture and siRNA interference

ECs and SMCs at Passage 4 to 6 were used for all experiments. Culture of ECs and SMCs in EC medium and SMC complete medium, respectively, at 37°C in humidified atmospheres containing 5% CO2 [19]. The Epsin 1 and Epsin 2 small interfering RNA were mixed with Lipofectamine to RNA-lipid complexes. As a control, ECs were washed with medium after initial stabilization in DMEM and 10%FBS for 7 h using scrambled siRNA under the same conditions.

### Mitochondrial staining and JC-1 staining

Mito Tracker: after the cells are treated, the cells are cultured in the plate or Petri dish, add Mito tracker working solution according to the instructions, and incubate at 37 °C for 30min. Remove the Mito Tracker working solution, wash it with PBS for 3 times, and add the cell culture medium [20]. JC-1: add JC-1 staining solution according to the instructions, and incubate in the cell incubator (Heraeus, Germany) at 37 °C for 20min. After incubation, the staining solution was removed and washed twice with JC-1 staining buffer [21]. Add the cell culture medium and observe under the microscope.

### Cell migration

The migration of ECs and smooth cells was analyzed by wound healing assay [17]. Cells were seeded in 6-well plates and evenly injured with the tip of the pipette. The standard photos of the wound area were taken through the microscope. After 24h, recorded the wound again. The distance of wound cells moving was calculated by subtracting the distance of 24h lesion edge from the distance of 0h lesion edge.

### Co culture model of ECs and SMCs

ECs were incubated with SMCs in Transwell chamber for 24 hours. SMCs were placed in lower chamber and ECs in the upper chamber (non-contact). After PBS washing for 3 times, the proliferation and migration of SMC were detected.

### Immunofluorescence

Cells were seeded on cover glass and soaked in PBS solution for 3 times, then perforated with 0.1% TritonX-100 [22]. After 30min, PBS was soaked for 3 times, sealed with goat serum for 2h, and subsequently, the prepared primary antibody solution was dripped onto the cells and incubated overnight in a 4°C refrigerator. Soaked in PBS for 3 times, incubated with fluorescent secondary antibody, incubated in dark for 2h, PBS was soaked for 3 times, and incubated with DPAI dye solution for 5min, then sealed. Observe with fluorescence microscope (Leica, Germany).

### Western blot

Total protein was extracted from EC and SMCs and then remove the supernatant and add RIPA to lyse the cells on ice for 30 minutes [19]. Remove the supernatant and measure the protein concentration. Adjust the protein concentration using RIPA. Prepare the protein at the appropriate concentration and add the corresponding volume of loading buffer. At 100°C in a water bath to denature the proteins. Prepare different concentrations of gel based on protein molecular weight according to the experimental plan. Stop electrophoresis when the proteins have separated to the appropriate position and remove the gel plate for membrane transfer. Block the PVDF membrane with 5% milk for 1 hour, rinse off the milk, and incubate with the prepared primary antibody solution overnight. Rinse the PVDF membrane with TBST 3 times and incubate for 1 hour with the secondary antibody solution Following three thorough rinses with TBST, images were acquired using an ImageQuant LAS500 instrument (GE Healthcare Life Sciences) in conjunction with the electrochemiluminescence-based Western blot analysis substrate.

### Epsin1-Epsin2-shRNA coating preparation

We use a layer-by-layer self-organization approach to form twelve layers of cation and anion layers on the surface of the stents, including Prs and plasmid with shRNA sequences [23]. The plasmid vector is pcDNA3.1-CMV-MCS-3flag-EF1-ZsGreen-T2A-Puro. A plasmid DNA (pDNA) vector encoding shRNA to target Epsin1 and Epsin2 (Epsin1-Epsin2-shRNA-pDNA) and scramble RNA obtained from HANBIO Solutions (China) were prepared. The PrS solution was prepared at a concentration of 1 mg/ml, while the pDNA solution was formulated to a concentration of 200 μg/ml. The substrates underwent initial immersion in the PrS solution for a period of 10 minutes, after which they were rinsed three times with HEPEs buffer solution, each rinse lasting one minute. Subsequently, the substrates were immersed in the DNA solution for an additional 10-minute duration, followed by another set of three thorough rinses. This process was repeated 12 times to form a 12 layer films: (PrS/Epsin1-Epsin2-shRNA-pDNA), and (PrS/ scramble RNA-pDNA). After the last rinse, the substrate was dried with a stream of nitrogen and stored in a desiccator at room temperature.

### *In vitro* shRNA transfection

The Epsin1-Epsin2-shRNA coating stents were placed in a six-well plate and 1 × 105/mL ECs were added to each well incubated at 37°C for 2 hours. Each well was filled with the same concentration of cell suspension after the stents were subjected to a 120° rotation along their longitudinal axis. After culturing for seven days, the Epsin1-Epsin2-shRNA coating stents were fixed using 4% paraformaldehyde for 30 minutes, then followed by an incubation of DAPI to stain the nuclei. In the laser confocal microscope (Leica Microsystems, Germany), DAPI-labeled nuclei of ECs were observed in blue fluorescence, while shRNA-transfected ECs were observed in green fluorescence.

### Surface characteristics

Use scanning electron microscopy (SEM) to examine the surface of the coating. The water contact angle on the surface was characterized using a water contact angle measurement device. Fix the nickel titanium alloy sample on the sample stage and place a drop of distilled water on the surface. After 2 seconds, measure the water contact angle. Use ImageJ software to detect the water contact angle of the sample on the surface [24].

### Blood compatibility experiment

The automatic coagulation analyzer is used to test the activated partial thromboplastin time (APTT) and prothrombin time (PT). After soaking each group of samples in PBS solution for 1 hour, add 500 μ Dilute platelet plasma and incubate at 37 ° C for 30 minutes. Then perform APTT and PT tests to analyze the coagulation time of the sample [25].

### *In vivo* stent implantation tests

The research animals are female domestic pigs weighing 30-35 kilograms. Selected animals were examined three days before implantation to verify clinical conditions. Every day for 3 days before surgery all animals received 75 mg clopidogrel and 300 mg aspirin during the follow-up period. The animals were intubated and mechanically ventilated after anesthesia with 25 mg/kg pentobarbital sodium. Eat and drink 24 hours before taking intervention measures. On the day of stent implantation, anesthetize pigs with ketamine (20mg/kg intramuscular injection) and penicillin (2mg/kg intramuscular injection). They continuously obtain 3 liters/minute of supplemental oxygen through an oxygen mask. After injecting 2% subcutaneous lidocaine, the right femoral artery was surgically exposed. Continuously monitor hemodynamics and surface electrocardiogram throughout the entire process. After intravenous injection of heparin (10000 units). Each pig had an Epsin1-Epsin2-shRNA coating stent or a void vector stent inserted randomly into his left carotid artery. A 6Fr diameter arterial sheath was inserted from the right femoral artery, and a 6Fr guiding catheter was inserted into the carotid artery. A balloon with 1.2:1 balloon to vessel ratio was placed at the distal end of the carotid artery guided by a 0.014-inch micro-guidewire. Under the roadmap, the stent was released. To determine whether there was a lumen obstruction, dissection of the vessel wall, embolism, stent displacement or distal vasospasm after stent implantation, an angiography was performed. Follow up angiography and tissue sampling were performed at one week and four weeks after stent implanting, respectively. At one week or four weeks, after injecting potassium chloride (20 mL) into the coronary artery, tissue samples were taken. the carotid artery with stent was removed, rinsed in PBS, and fixed with 3% glutaraldehyde. As previously report report [26]).The vessels were stained with HE, IH and vascular wall injury were observed under a light microscope (Leica, Germany). ImageJ software was employed for statistical analysis of the stent area and lumen area, with the intimal area calculated using the formula (stent area - lumen area). The percentage of stenosis area was determined by applying the formula: (1 - lumen area/ stent area) ×  100%.

### Statistical analysis

Statistical analysis was carried out using Graphpad Prism v9.0 software. A one-way analysis of variance (ANOVA) was applied to all datasets. For the assessment of differences between groups, Tukey’s multiple comparison test was utilized. A p-value less than 0.05 was considered statistically significant.

## Results

### Epsin regulated neointimal formation in injured carotid arteries

**Fig 1:**
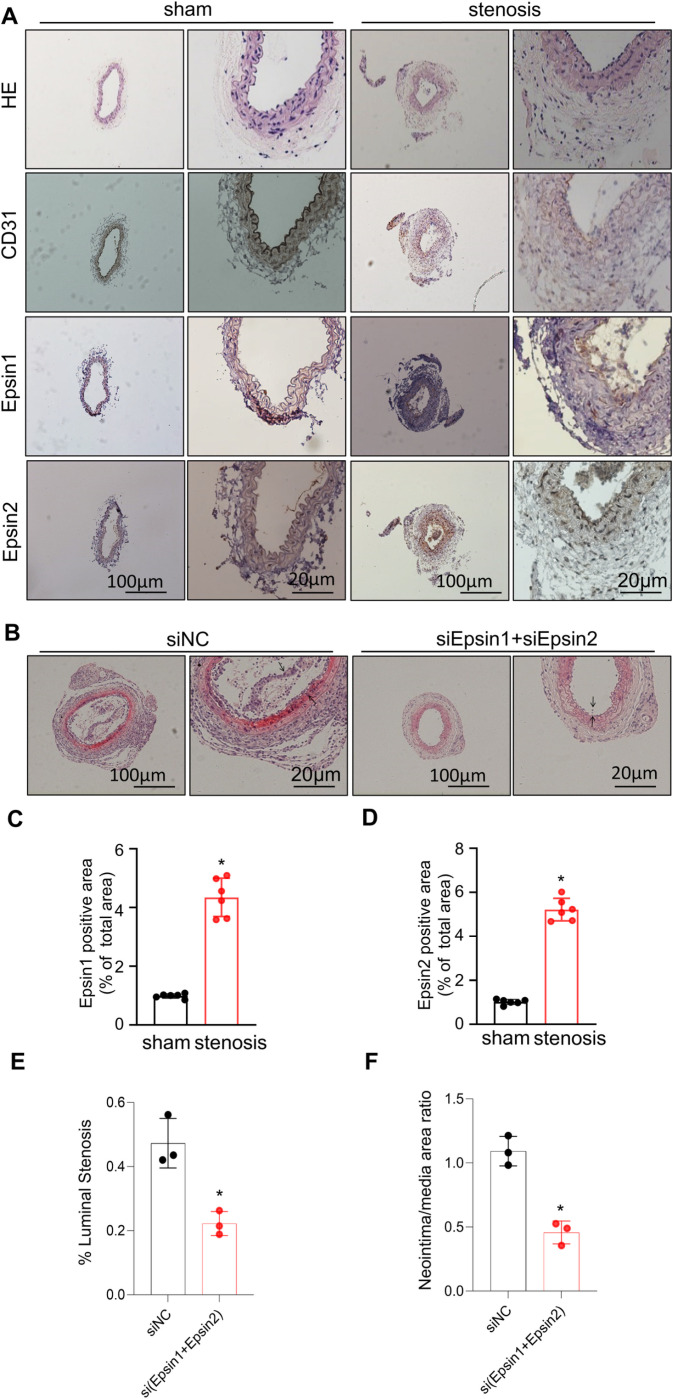
Expression of Epsin in Carotid Arteries of Mice in Each Group after 7 Days. (A) Immunohistochemical staining with specific anti‐CD31 Epsin1 and Epsin2 antibodies in mice carotid arteries. The relative quantification of Epsin protein expression. (B) and (C) Image J software was applied to evaluate protein expression according to the grayscale values. (D) H&E‐stained carotid arteries treated with sh(Epsin1 + Epsin2) or shNC at 7 days after injury. (E) and (F) Quantification of neointimal area and neointima/media ratio of carotid arteries treated with sh(Epsin1 + Epsin2) or shNC. t-test, n = 6 in each group. * : P < 0.05 versus the Sham group.

The carotid artery ligated group showed significant thickening of the intima and media compared with the sham group, as well as proliferation of ECs and SMCs (Fig 1A). Immunohistochemical analysis revealed that Epsin1 and Epsin2 were almost not expressed in the sham group, the expression of Epsin1 and Epsin2 were significantly upregulated in the intima and media of the IH group (Fig 1A, B, C). H&E staining showed that the Epsin knockdown significantly inhibit the intimal thickening. The neointimal area of arteries with Epsin knockdown was significantly reduced than that with shNC at 7 days. Accordingly, the ratio of neointima to media area was significantly lower in sh(Epsin1 + Epsin2)‐treated arteries than negative control arteries (Fig 1D, E, F).

### Knockdown of Epsin promoted proliferation and migration in ECs

**Fig 2:**
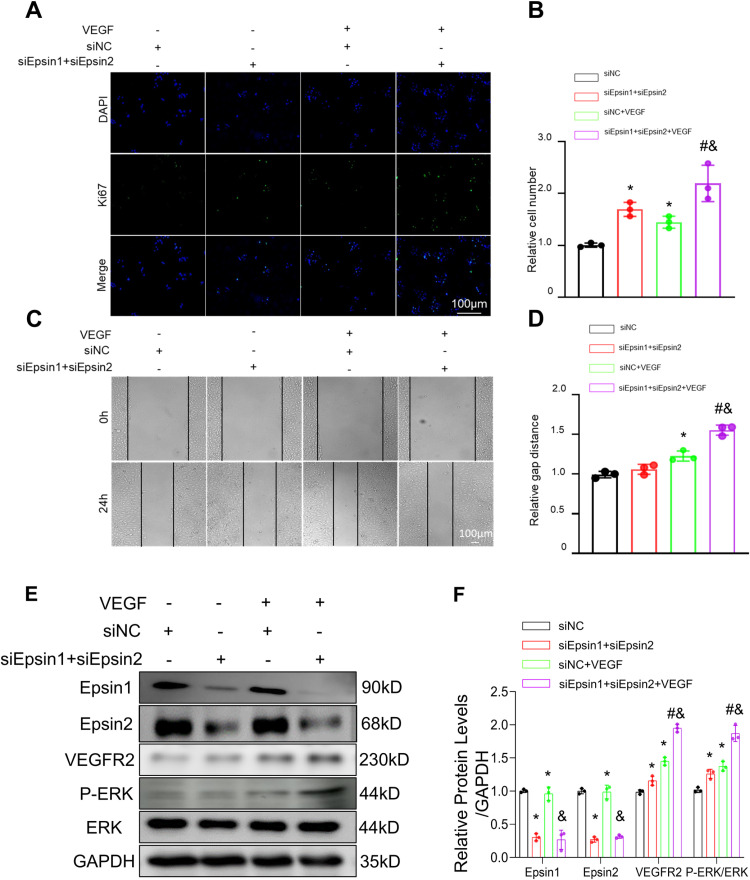
The effect of knocking down Epsin on EC proliferation and migration. (A) and (B) Ki67 incorporation assays of EC. Representative immunofluorescence of Ki67 (green) and DAPI (blue). percentages of Ki67 incorporated EC. (C) and (D) Scratch‐wound assays showed the effect of VEGF on the migration of EC infected with siEpsin1 + siEpsin2. (E) and (F): expression levels of Epsin1, Epsin2, VEGFR2 and Erk were determined by Western blotting using the appropriate antibodies. GAPDH served as a loading control. GAPDH served as a loading control. t-test, n = 3, in each group. * : P ＜ 0.05 vs. sinormal control (NC); #: P ＜ 0.05 vs. siEpsin1 + siEpsin2; &: P ＜ 0.05 vs. siNC+VEGF.

ECs spreading is critical for the reendothelialization after stent implantation. We aimed to investigate the impact of Epsin knockdown on the proliferation and migration of ECs. Proliferating cell nuclear antigen Ki67 was used to assess cell proliferation (Fig 2A, B), and a scratch test was employed to measure cell migration (Fig 2C, D). Knockdown of Epsin significantly increased the number of Ki67 positive ECs, indicating enhanced cell proliferation compared to control cells (Fig 2A, B). VEGF promotes the proliferation and migration of vascular endothelial cells, which is necessary for blood vessel formation. After 24-hours incubation with VEGF concentration of 100 μL, knockdown of Epsin resulted in a longer relative migration distance compared to control cells (Fig 2C, D). The expression of VEGFR2 and activation of ERK downstream were higher in cells with Epsin knockdown (Fig 2E, F).

### Knockdown of Epsin enhanced endothelial proliferation and migration via protecting mitochondrial function

**Fig 3:**
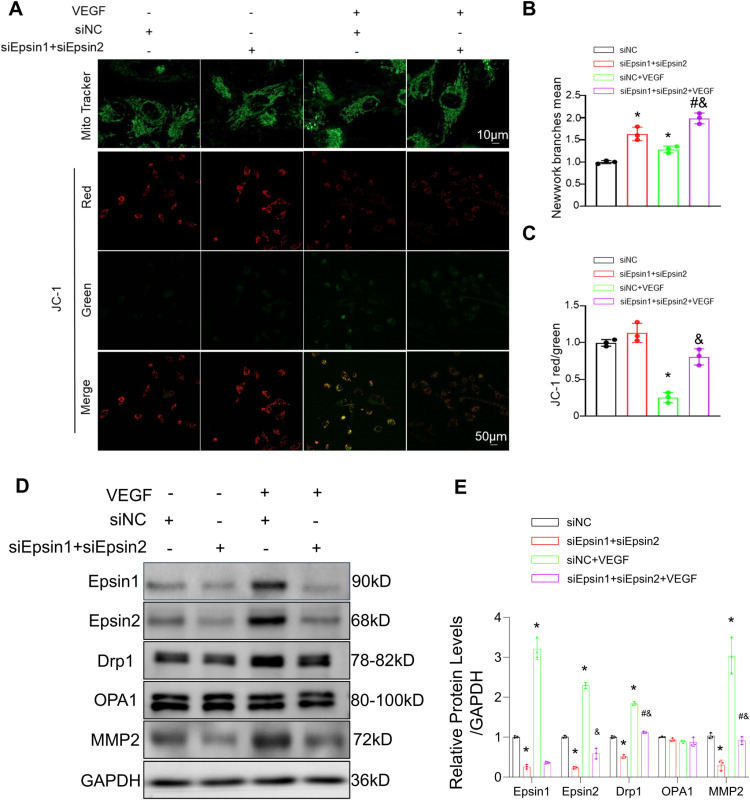
In vitro, Epsin regulates EC mitochondrial dynamics, membrane potential, and function. **(A)** MitoTracker Green staining showed the mitochondrial morphology of EC after 24 hours of treatment. (B) and (C) mitochondrial membrane potentials of EC were evaluated using JC-1 staining after treating for 24 h, J-aggregates (red) and monomers (green). (D) and (E) expression levels of Epsin1, Epsin2, Drp1 and Opa1 were determined by Western blotting using the appropriate antibodies. GAPDH served as a loading control. t-test, n = 3, in each group. * : P ＜ 0.05 vs. sinormal control (NC); #: P ＜ 0.05 vs. siEpsin1 + siEpsin2; &: P ＜ 0.05 vs. siNC+VEGF.

To gain insights into the potential mechanism by which Epsin promoted the proliferation of ECs, we conducted experiments to assess mitochondrial content and function. MitoTracker Green and JC-1 mitochondrial staining were employed to study these changes. Our findings revealed that knockdown of Epsin significantly increased the mitochondrial content of ECs, and an expanded mitochondrial surface area indicating heightened energy demand within the cells. (Fig 3A, B). After VEGF stimulation, ECs mitochondrial homeostasis was disrupted, and when we knocked down Epsin, this negative change was suppressed. (Fig 3A, C). WB indicated that knockdown of Epsin, down regulated the expression of Drpl without affecting the expression of OPAl (Fig 3D, E).

### Epsin knocked down in ECs inhibited the proliferation and migration of co cultured SMCs

**Fig 4:**
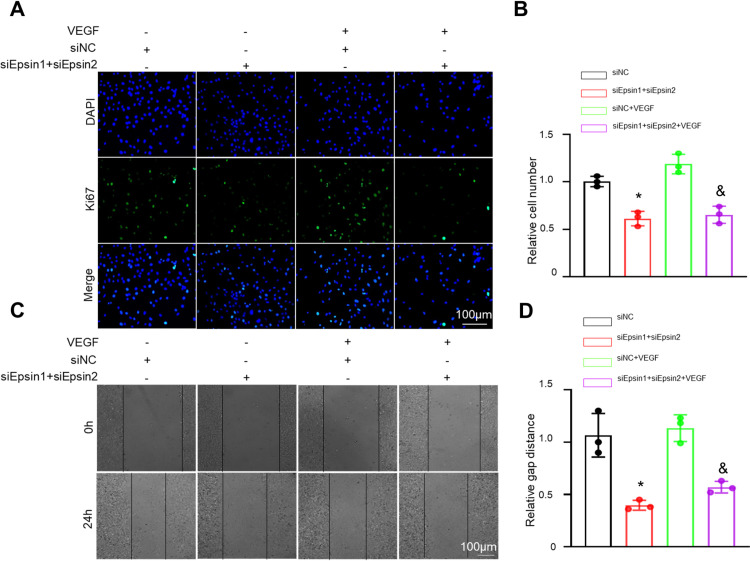
The impact of Epsin knockdown in ECs on the proliferation and migration of co-cultured SMCs. (A) and (B) Ki67 incorporation assay of SMCs, treated as in A for 48 hours. representative immunofluorescence of Ki67 (green) and DAPI (blue). percentages of Ki67 incorporated EC. (C) and (D) migration ability assessed by wound healing. t-test, n = 3, in each group. * : P ＜ 0.05 vs. sinormal control (NC); #: P＜ 0.05 vs. siEpsin1 + siEpsin2; &: P ＜ 0.05 vs. siNC+VEGF.

MMP2 is involved in adventitia degradation, which is crucial for the regulation of vascular SMCs muscle cell proliferation, migration, and development of vascular IH. We observed that knockdown of Epsin had a downregulating effect on matrix metalloproteinase 2 (MMP2), an important protein known to promote the proliferation and migration of SMCs (Fig 3D, E). Notably, the relative migration distance of SMCs co-cultured with Epsin knocked down ECs was significantly weaker compared to control group (Fig 4C, D). Furthermore, Ki67-positive cells, as an indicator of cellular proliferation, was lower in the co-culture with Epsin knocked down ECs (Fig 4A, B), indicating that knocked down of Epsin indirectly inhibited the proliferation and migration of SMCs by downregulating the expression of MMP2 in ECs.

### The Epsin1-Epsin2-shRNA stent had feasible transfection efficiency and effectively inhibited IH *in vivo*

**Fig 5:**
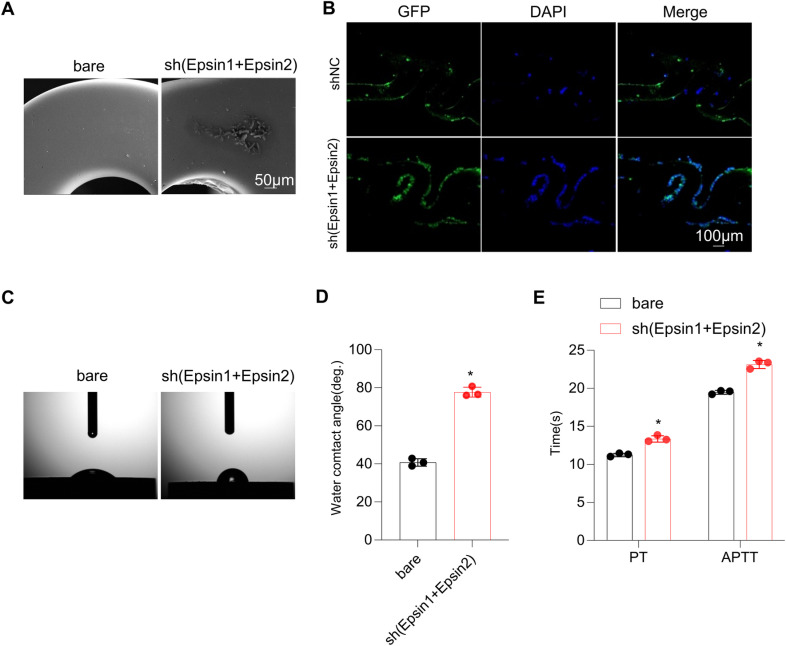
Transfection efficiency, surface characteristics. (A) A scanning electron microscope results showed surface of bare-metal stents and a collagen layer of shEpsin1 + siEpsin2 stents. (B) photographed from a confocal laser scanning microscope, cells on the stent were transfected. DAPI stained nuclei of the ECs on stent were blue and shRNA-transfected cells were green. Scale bars are 500µm SEM and water contact angle analysis. (C) and (D) Representative images of water contact angle testing on different surfaces. (E) Activated partial thromboplastin time (APTT) and prothrombin time (PT) measurements.

**Fig 6:**
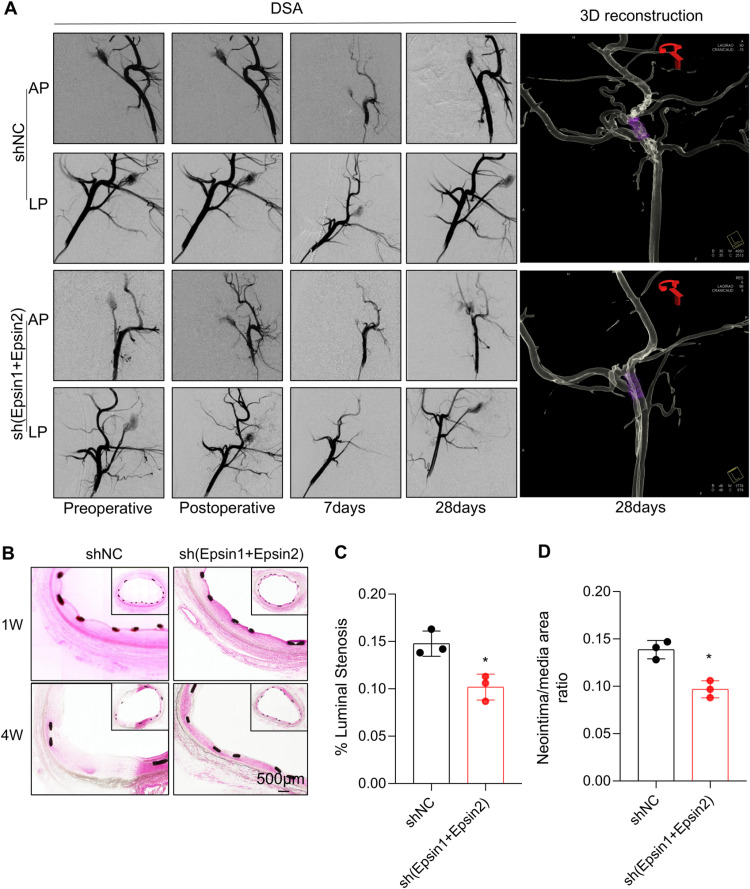
Carotid artery implantation results of Epsin-eluting stent. (A) Representative Digital substraction angiography images after stents implantation in swine carotid arteries show no thrombus and stent displacement at perioperative period. AP: anteroposterior position, LP: lateral position. n = 3.(B),(C) and (D) H&E staining (×200) showed IH in the shnormal contrast (NC) stent group and the sh(Epsin1 + Epsin2) void vector group * : P ＜ 0.05 vs. shNC group; n = 3.

PrS with pDNA were entirely coated on stents, we found that the metal surface is smooth without accessories, and the surface coating is uniform (Fig 5A). ECs were seeded on the stent surface, proliferated along the surface of stents, and were almost completely wrapped the stents and distributed evenly, green fluorescence indicating that they have been transfected (Fig 5B). The proportion of endothelial wrapping area and ECs count of Epsin1-Epsin2-shRNA eluting stent was significantly increased (Fig 5B). Water contact angle measurement is used to evaluate the hydrophilicity of material surfaces. The smaller the contact angle, the better the hydrophilicity (Fig 5C). The coated stent group exhibits poor hydrophilicity and is less likely to adhere platelets. Simultaneously using APTT and TT methods to evaluate multilayer films (Fig 5E). There were significant differences in APTT and TT results between the bare stent group and the coated stent group. Compared with bare scaffolds, multilayer membranes have better anticoagulation performance. In-stent intimal hyperplasia was examined by angiographic and morphological analysis in a swine carotid artery model. The preoperative and postoperative angiogram in the same pig showed that stent was implanted successfully and no signs of stent malposition or acute embolism (Fig 6A). At 7 days and 28days post-surgery, angiography confirm that the lumen was patency and unobstructed, no stent migration (Fig 6A). HE staining showed that monolayer ECs covered on the Epsin1-Epsin2-shRNA coated stents at 1 week after implantation, while the control stents were directly exposed to the blood (Fig 5B). Compared with the control group, Epsin1-Epsin2-shRNA eluting stented group showed low intimal hyperplasia. In addition, the intima-to-media ratio was also lower than that of the control stent group (Fig 6C, D).

## Discussion

In order to overcome the disadvantage that drug coated scaffolds damage endothelial function, our study coated protamine and plasmids with Epsin1 and Epsin2 shRNAs on the stent in a layer-by-layer self-organization manner. Inhibition of Epsin expression protects endothelial cell function while inhibiting carotid intimal hyperplasia in both mice and porcines. Our study has generated the following findings: In vitro experiments, knocking down Epsin promotes ECs proliferation and migration, while enhancing ECs survival and maintaining mitochondrial homeostasis. Knocking down Epsin *in vivo* promotes early endothelialization after stent implantation, while effectively preventing IH. *In vitro* experiments, knocking down Epsin promoted endothelial cell proliferation and migration, while enhancing endothelial cell survival and maintaining mitochondrial homeostasis. Further study of the mechanism of Epsin on mitochondrial content and function. ECs proliferation and migration are the first steps in reendothelialization after stent implantation [27]. DES have significantly reduced restenosis, however, they have concurrently been found to be associated with long-term thrombosis of the stents. An alternative strategy to reduce stent thrombosis may be to reduce restenosis while promoting endothelialization [28]. Through the measurement of water contact and APTT and PT, we found that the coating reduces the hydrophilicity of the scaffold surface and has a certain anticoagulant effect, while the hydrophobic surface hinders the close contact between the substrate and the corrosive environment while effectively resisting platelet adhesion [29]. Our experiments found that Epsin had a specific dual effect of promoting endothelial and inhibiting smooth muscle in vitro. In this report, Surface-mediated gene delivery layer-by-layer membranes containing Epsin-shRNA-pDNA vectors can reduce IH within stents by silencing Epsin expression. The shRNA pDNA vector showed significant transfection effects in ECs. We implanted the Epsin1-Epsin2-shRNA elution stent, and at 1 week after stent implantation, the Epsin1-Epsin2-shRNA functional coating formed a monolayer of ECs on the stent, while the control group was directly exposed to blood. One month later, the IH in the Epsin1-Epsin2-shRNA elution functional coating stent group was significantly lower than the control. The reduction of local Epsin expression after stent implantation may promote early reendothelialization after stent implantation and inhibit the proliferation of local neointima.

ECs have the function of inhibiting platelet aggregation and thrombosis. By forming a complete ECs layer as soon as possible, the risk of thrombosis can be reduced and the chance of stent thrombosis can be reduced. Furthermore, it can make SMCs directly exposed to various mitogens in the serum, which will stimulate the proliferation of SMCs [30]. ECs can produce a range of anti-inflammatory and antioxidant substances, which help maintain the healthy state of the blood vessel wall. The early formation of an ECs layer can alleviate inflammatory responses and oxidative stress, preventing damage and loss of blood vessel walls, and restoring normal structure and function of blood vessel around the stent. Although both groups showed reendothelialization after four weeks, the intimal proliferation in the experimental group was significantly reduced. Therefore, it was clear that the coated stent in the experimental group played a role in preventing restenosis by protecting ECs and inhibiting SMC proliferation. Further in-depth research was needed to determine the underlying mechanisms behind these different processes. Moreover, protecting the normal function of ECs, which was important in proliferative vascular diseases such as restenosis following carotid artery injury [31].

Indeed, normal mitochondrial morphology was fundamentally important for sustaining mitochondrial functionality [32], mitochondrial dysfunction leaded to accelerate the process of cellular apoptosis, accompanied by oxidative stress and impaired cellular metabolism [33]. Mitochondrial fusion requires regulation by OPA1. Mitochondrial fission control requires Drp1, and mitochondrial dynamics play an important role in maintaining normal cellular function, regulating mitochondrial metabolism, promoting mitochondrial fragmentation, activating mitochondrial autophagy, regulating mitochondrial outer membrane permeability, and participating in cell apoptosis [34,35]. Due to its dynamic nature, mitochondria function was closely linked to the balance between fusion and fission. It has been demonstrated that an excessive degree of mitochondrial fission, regulated by Drp1, can give rise to oxidative stress, disrupt energy supply mechanisms, and ultimately result in cell death [36]. Epsin 1 and 2 played a novel role in altering mitochondrial content and function in ECs. Endothelial mitochondrial fission is one of the major mechanisms leading to aberrant vascular proliferation, but its underlying molecular mechanisms are still elusive. It plays a pivotal role in widely accepted “endothelial damage” model [37,38]. Our results showed that knockdown of Epsin reduced the expression of Drp1 in ECs (Fig 5A, B), which was associated with less mitochondrial fission, altering mitochondrial content. It maintains mitochondrial membrane potential homeostasis and preserves mitochondrial activity, maintaining the relative stability of ECs. Our observations suggested that knockdown of Epsin promoted the migration and proliferation of ECs, potentially by modulating the content and function of mitochondria. These findings shed light on the underlying mechanisms by which Epsin impacted themigration, and matrix invasion among different cell types, such as tumor cells, fibroblasts, and smooth muscle cells in the vascular system [39–41]. There are multiple mechanisms through which MMP2 can promote SMC migration and proliferation [42]. Activated MMP2 degrades type IV collagen, elastic fibers, and basement membrane, disrupting the inhibitory mechanisms between SMCs and extracellular matrix (ECM) [43].SMC migration and proliferation may be further promoted through integrin FAK signaling by remodeling ECM components [44,45]. In addition, MMP2 relieved connections between SMCs by cleaving N-cadherin, which promoted their migration and proliferation [46,47]. MMP2 expression may also play an important role in facilitating the mobilization and release of cytokines and growth factors within the extracellular matrix, thereby enhancing the activity of SMCs during neointimal formation [48]. The reduction of Epsin promoted reendothelialization, enhances ECs activity, and inhibited smooth muscle cell proliferation by reducing metalloproteinase secretion when co-cultured with smooth muscle cells. Previous studies have found that Epsin has multiple functions and is highly specific in targeting protein ECs migration and proliferation and may have implications for therapeutic interventions aimed at promoting tissue repair and angiogenesis.

In addition, we found that Epsin knockdown indirectly inhibited SMCs proliferation and migration by downregulating MMP2 expression in ECs. In SMC proliferation, migration, and intimal formation, matrix metalloproteinases (MMPs) degrade extracellular matrix (ECM) [49]. The key enzyme in this remodeling process, MMP2, promoted cell proliferation, s. Its functions varyed in pathological and physiological states and in different cells. Episn participated in the whole process of atherosclerosis formation and progression through a variety of signal pathways, including lipid uptake, atherosclerotic plaque, plaque rupture, and was associated with inflammation. At the same time, it aggravated the adverse prognosis of atherosclerosis related diseases. The decrease of Epsin regulated the internalization of IP3R1 and VEGFR2 in ECs [50], inhibited the transformation of vascular ECs into mesenchyme, and promoted endothelial proliferation and migration. Epsin in macrophages promoted the degradation of ABCG1 and LRP-1 through lysosomes [14,27], promoted lipid uptake, blocked cholesterol outflow and reversed cholesterol transport, and enabled macrophages to transform to the pro inflammatory direction, thereby reducing the necrotic core in atherosclerotic plaque, reducing the risk of atherosclerotic plaque rupture flowing to the brain and heart, causing stroke and coronary heart disease. At the same time, Epsin alleviated inflammatory reaction in atherosclerotic lesions and slowed down the development of atherosclerosis. Epsin loss reduced adhesion molecules and inflammatory factor MCP-1 expression in ECs [51]. In mice with endothelial Epsin deficiency, oxidative stress and endoplasmic reticulum stress, important components of these inflammations were significantly reduced. Mechanistically, Epsin modulated inflammatory signaling pathways and augment the activation of ECs through its interaction with interacting with components of the Toll like receptor and NFκB signaling pathway to some extent [51]. From a clinical perspective, reducing inflammation in patients after stent implantation can significantly decreased the risk of restenosis in human stents. Although we have gained some understanding of this field, further research is still needed to answer questions about the specific mechanisms.

Our current animal experiments only describe morphological differences. In the future, further validation will be conducted on the expression of relevant molecules to confirm the role of Epsin. In addition, we should conduct more in-depth research on the molecular pathways involved. Currently, it has been found that mice lacking Epsin1 and Epsin2 die on the 10th day of embryonic development and exhibit abnormal vascular phenotypes [15]. However, the lack of endothelial Epsin1 and Epsin2 in adulthood does not affect normal blood vessels. But we still need to further investigate the long-term effects and potential side effects or complications of long-term inhibition of Epsin on vascular health and restenosis prevention in animal experiments, and compare it with currently available drug-eluting stents or other standard therapies for preventing restenosis. And further collect clinical data to support its effectiveness in the human body. In summary, although this article proposes a new and potentially effective method for preventing stent stenosis, it has some limitations, including a lack of clinical data, incomplete exploration of side effects, and unknown long-term effects. Further research is needed to validate these findings in clinical settings and fully understand the mechanisms and safety of Epsin inhibition.

## Conclusion

In summary, our results suggest that down-regulated Epsin can effectively prevent IH, while promoting EC proliferation and reendothelialization during the initial stage of stent implantation. In addition, Epsin down-regulating can improve cell viability and maintain EC homeostasis by inhibiting abnormal mitochondrial fission, preventing normal EC function from being damaged, and reducing excessive proliferation of smooth muscle cells caused by endothelial stimulation. Therefore, targeting Epsin activity may be an effective target for preventing restenosis after stent implantation in patients.

## Supporting information

S1 Raw images Western blot raw data for Fig 2D and Fig 3D. (PDF)

## Abbreviations

shRNA: short hairpin RNA
IH: intimal hyperplasia
CCA: common carotid artery
Drp1: dynamin-related protein 1
ECA: external carotid artery
Epsin1: EPS-15-interacting protein 1
Epsin2: EPS-15-interacting protein 2
ERK: extracellular regulated protein kinases
GAPDH: glyceraldehyde-3-phosphate dehydrogenase
HE: hematoxylin-eosin
ICA: internal carotid artery
MMP2: matrix metalloproteinase
OPA1: mitochondrial dynamin like GTPase
PBS: phosphate buffer saline
TBST: tris-buffered saline and tween
Prs: Protamine sulfate
ECM: extracellular matrix.
