# Supplementary figures and images for: Epsin bioactive coating reduced in-stent intimal hyperplasia by promoting early phase reendothelialization and inhibiting smooth muscle cell proliferation
Source: PLoS One. 2025 Mar 25;20(3):e0318019. doi: 10.1371/journal.pone.0318019 (PMC11936285; doi:10.1371/journal.pone.0318019)

Fig2D

|                   |   |   |   |   |
|-------------------|---|---|---|---|
| VEGF              | - | - | + | + |
| siNC              | + | - | + | - |
| siEpsin1+siEpsin2 | - | + | - | + |

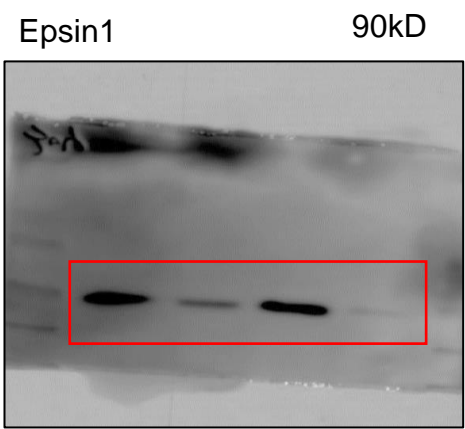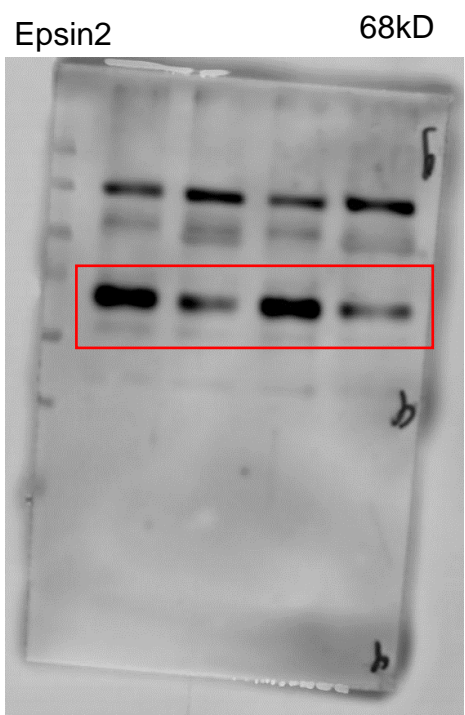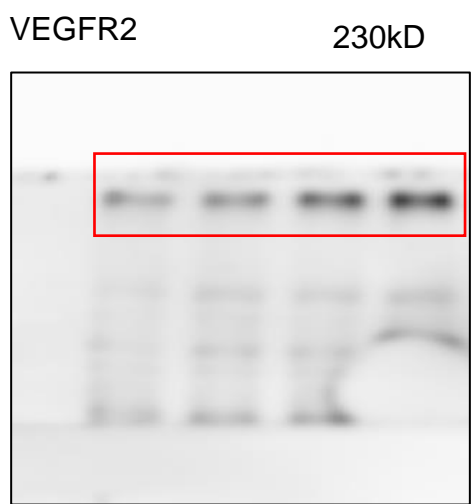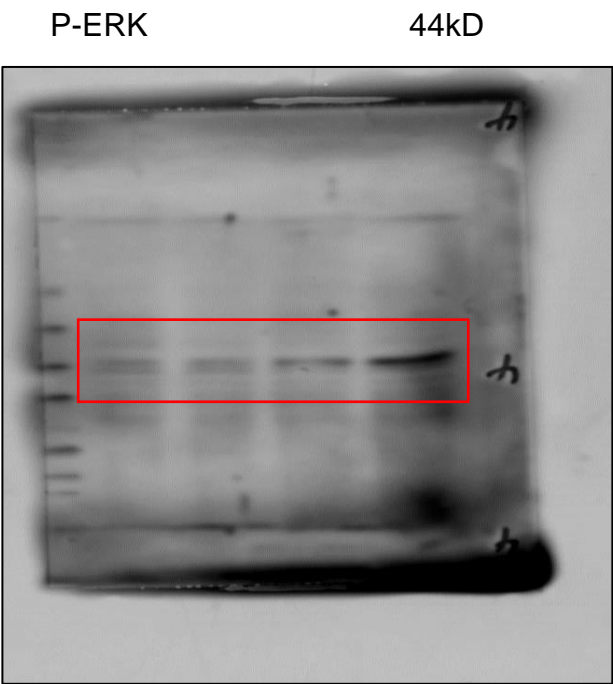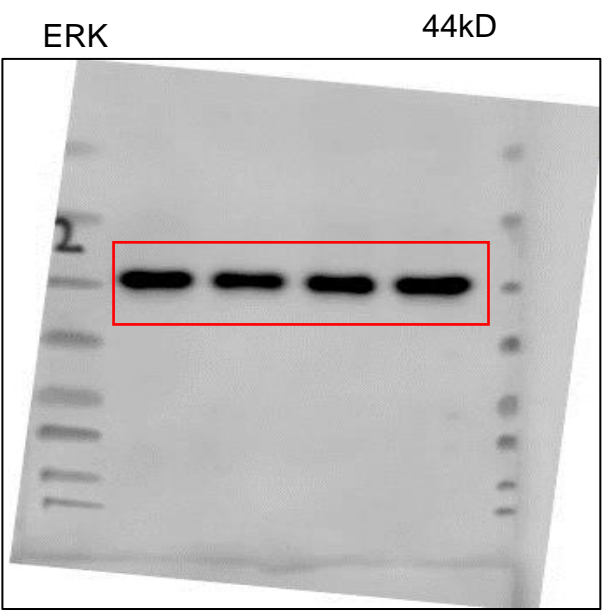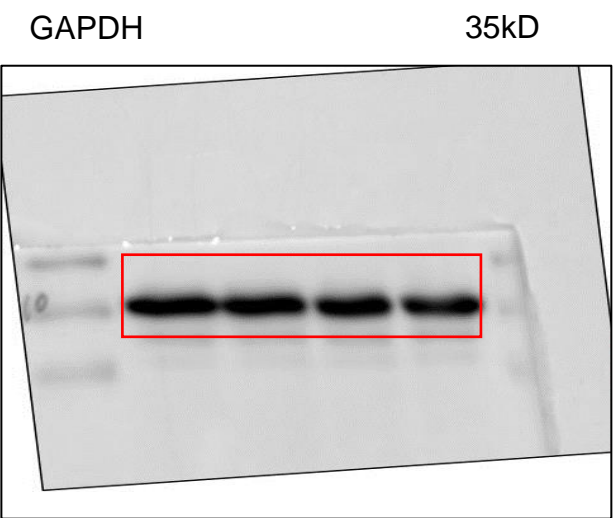

Fig3D

|                   |   |   |   |   |
|-------------------|---|---|---|---|
| VEGF              | - | - | + | + |
| siNC              | + | - | + | - |
| siEpsin1+siEpsin2 | - | + | - | + |

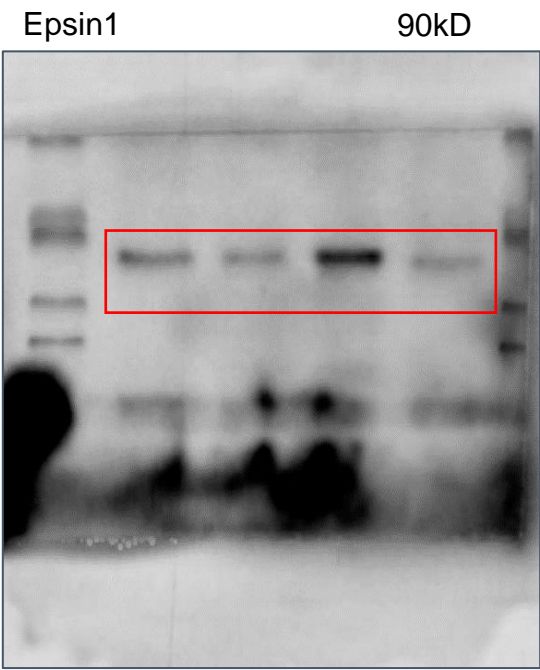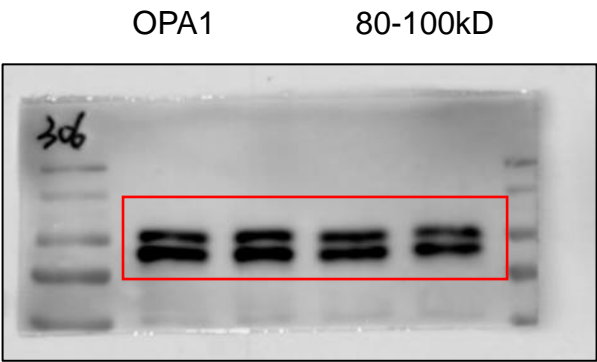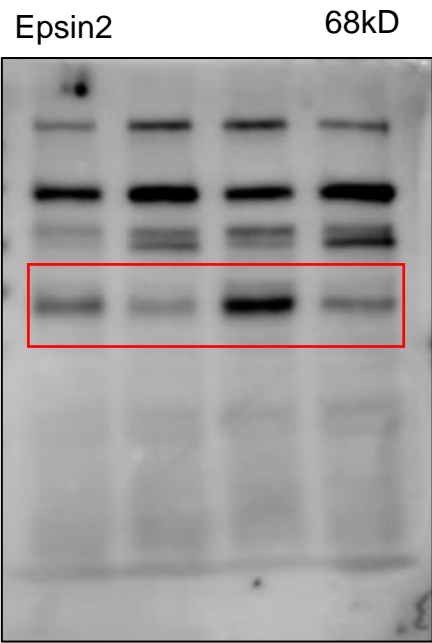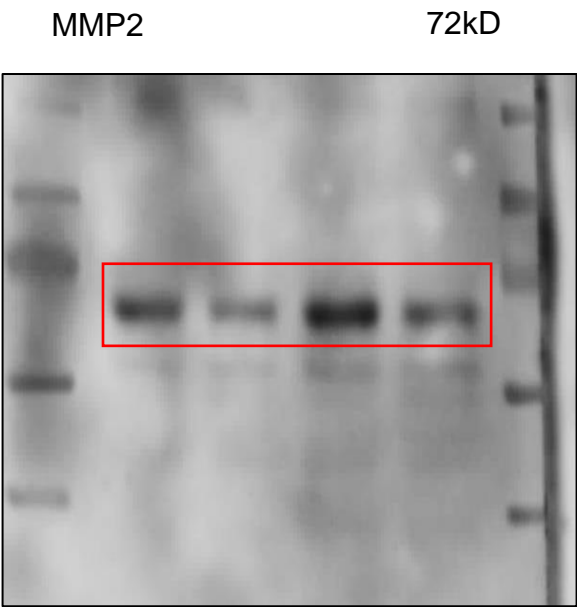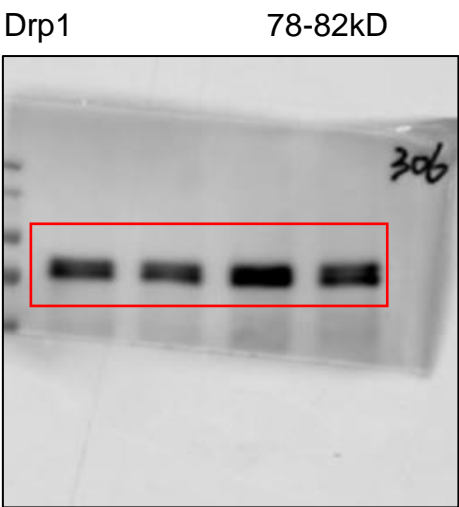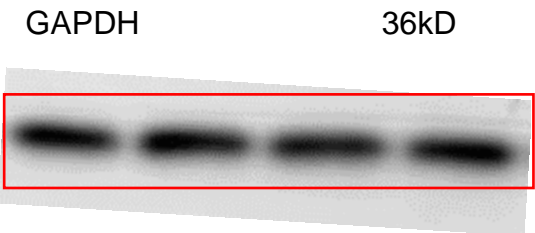

Supplement: S1 Raw images — (PDF) [file pone.0318019.s001.pdf]
